# The dynamic response of the Arabidopsis root metabolome to auxin and ethylene is not predicted by changes in the transcriptome

**DOI:** 10.1038/s41598-019-57161-9

**Published:** 2020-01-20

**Authors:** Sherry B. Hildreth, Evan E. Foley, Gloria K. Muday, Richard F. Helm, Brenda S. J. Winkel

**Affiliations:** 1 Department of Biological Sciences, Virginia Tech, Blacksburg, VA United States of America; 2 Department of Biochemistry, Virginia Tech, Blacksburg, VA United States of America; 3 Department of Biology and Center for Molecular Signaling, Wake Forest University, Winston-Salem, North Carolina United States of America

**Keywords:** Biochemical networks, Plant sciences, Auxin

## Abstract

While the effects of phytohormones on plant gene expression have been well characterized, comparatively little is known about how hormones influence metabolite profiles. This study examined the effects of elevated auxin and ethylene on the metabolome of Arabidopsis roots using a high-resolution 24 h time course, conducted in parallel to time-matched transcriptomic analyses. Mass spectrometry using orthogonal UPLC separation strategies (reversed phase and HILIC) in both positive and negative ionization modes was used to maximize identification of metabolites with altered levels. The findings show that the root metabolome responds rapidly to hormone stimulus and that compounds belonging to the same class of metabolites exhibit similar changes. The responses were dominated by changes in phenylpropanoid, glucosinolate, and fatty acid metabolism, although the nature and timing of the response was unique for each hormone. These alterations in the metabolome were not directly predicted by the corresponding transcriptome data, suggesting that post-transcriptional events such as changes in enzyme activity and/or transport processes drove the observed changes in the metabolome. These findings underscore the need to better understand the biochemical mechanisms underlying the temporal reconfiguration of plant metabolism, especially in relation to the hormone-metabolome interface and its subsequent physiological and morphological effects.

## Introduction

The phytohormones, auxin and ethylene, regulate physiological and developmental processes in plants, synergistically controlling some responses, while antagonistically regulating others^1,2^. The morphological changes effected in roots by these two hormones are well characterized, with both hormones inhibiting primary root elongation and stimulating root hair formation. In contrast, the hormones have opposite effects on lateral root development, with auxin stimulating and ethylene inhibiting this process^1,3,4^.

Substantial progress has been made in elucidating the auxin and ethylene signaling and transcriptional response pathways and the processes that control the homeostasis and intracellular movement of these two distinct, but highly interactive and interdependent hormone (reviewed in^5–7^). Auxin, most commonly found in plants in the form of indole-3-acetic acid (IAA), is an aromatic molecule synthesized predominantly from tryptophan, while ethylene is a simple gaseous hydrocarbon (C2H4) derived from methionine via 1-aminocyclopropane-1-carboxylic acid (ACC). In Arabidopsis, ethylene and auxin responses are initiated by binding of the hormones to their receptors, with ETR1 and TIR1 being the best characterized members of ethylene and receptor families^8^. Binding to these receptors initiates a cascade of well-defined processes that in both cases involves inhibition of negative regulators, but via very different mechanisms. The signaling cascades ultimately results in transcriptional control of a variety of target genes, with cross talk occurring through activation of promoters containing both auxin and ethylene regulatory elements and formation of transcription factor complexes. Secondary crosstalk occurs through genes that respond to one of the hormones but then regulate the synthesis, transport, or response pathways of the other. Auxin gradients are critical to driving directed development in plants and are established through a combination of homeostasis mechanisms that include *de novo* synthesis, conjugation to sugars and amino acids, and oxidation^7^, as well as polar auxin transport mediated by PIN-FORMED (PIN)^9^ and ABCBAUX1/LIKE-AUX1^10^ transporters. Auxin transport is regulated by PIN protein localization as well as the activity of specialized metabolites such as the flavonol, quercetin. Ethylene, which is a gas, is synthesized at or near its site of action, and is able to move between cells by diffusion.

To identify downstream targets of the auxin and ethylene signaling machinery that drive changes in growth and development, transcriptome analyses of the immediate response of Arabidopsis seedling roots to auxin and ethylene application were previously undertaken at high temporal resolution^11,12^. These time courses comprised eight time points over a 24 h period, which were overlaid on time-matched developmental controls. This large-scale effort identified clusters of genes exhibiting similar kinetic patterns that, together with subsequent genetic analyses, provided new insights into the immediate impact of these hormones on global gene expression. This included identification of cell wall remodeling enzymes that participate in auxin-stimulated lateral root developmentt^11^. Much less is known about metabolic enzyme targets that mediate metabolite synthesis, transport, and function to produce specific outcomes at the cellular and organismal levels.

The current study examined the root metabolome over the same high-resolution time course and with the same global approach used for the transcriptome studies. This made it possible to ask whether changes in the profile of metabolites in these tissues were correlated with changes in expression of genes encoding metabolic enzymes, or whether other mechanisms may contribute to the early response to hormone exposure. Prior studies examining the influence of auxin and ethylene on root metabolism have generally focused on a specific class of metabolites, a single time point, or mutants with altered abilities to respond to or synthesize auxin and/or ethylene (e.g.,^13–15^). In an effort to match the global approach undertaken in the transcriptome studies, the metabolomics analysis presented here utilized two modes of chromatographic separation, reversed phase (RP) and hydrophilic interaction chromatography (HILIC), both performed in positive and negative ion modes, generating an overview of the chemically-diverse root metabolome at each time point. The resulting data files were processed into four datasets, using untargeted metabolomics methods for unsupervised discovery of features that changed in response to hormone treatment. Fragmentation analysis was performed on features of interest and the resulting spectra were compared to those published in metabolite databases to obtain putative identifications of compounds that exhibited substantial changes relative to time-matched controls across the root metabolome, and included sugars, amino acids, glucosinolates, phenylpropanoids, oligolignols, phospholipids, and indole-containing compounds^16–18^. These experiments provide evidence of a rapid, transient change in the root metabolome primarily involving a small subset of specialized metabolites. Based on comparison with microarray data compiled for a parallel time course, the observed alterations in the metabolome generally did not appear to result from changes in gene expression, but rather from reconfiguration of existing biochemical and/or transport networks. Moreover, despite extensive crosstalk between auxin and ethylene at the transcriptional level and in mediating developmental and stress responses^19–21^, the immediate effects of the two hormones on the root metabolome were quite distinct, both with respect to timing and the specific metabolites that were altered.

## Results

### Experimental design and metabolomic data analysis

The goal of this study was to examine the root metabolome in the first 24 h following exposure to the endogenous auxin, IAA, or the ethylene precursor, ACC. Experiments were carried out under conditions that paralleled earlier transcriptome studies so as to also enable exploration of cause and effect relationships at these two levels of cellular phenotyping^11,12^. Seedlings were grown for 5d on nylon filters overlaid on MS-sucrose-agar. The filters were then moved to medium containing 1 µM IAA or 1 µM ACC. Root samples were collected at 0 (initiation), 0.5, 1, 2, 4, 8, 12 and 24 h after transfer, with biological replicates obtained from three independent experiments. The resulting samples (24 each for IAA-treated, ACC-treated, and untreated controls) were analyzed using four different LC/MS methods that utilized two modes of chromatographic separation (reversed phase and HILIC), each with two mass spectrometer polarities (positive and negative). These complementary approaches maximized coverage and provided a robust sampling of the metabolome. Each sample was analyzed in technical triplicate, a total of 864 injections. A representative chromatogram from each type of LC-MS analysis is shown in Fig. S1.

The raw chromatograms were processed into a data matrix to allow for characterization of changes in the metabolome across hormone treatments and time. Data from each of the four analyses modes were analyzed separately, each generating between 400 and 1600 features, defined as an exact mass/retention time pair (EMRT) and its corresponding peak area. The full datasets are provided as supplementary material (Datasets S1–S4). Redundant features resulting from naturally-occurring isotopes, adducts, and in-source fragmentation were identified and merged using RamClustR^22^ to generate a deconvoluted data set. Features exhibiting robust changes in response to hormone exposure were defined as those that displayed at least a two-fold difference between treated and untreated tissues in all three biological replicates. The results of this processing are summarized in Table 1. Overall less than 15% of the features detected exhibited a two-fold difference.

**Table 1:** Summary of features discovered by UPLC-MS analysis of Arabidopsis roots.

MS Method	Total identified by XCMS	Following deconvolution by RamClustR	Different following IAA treatment	Different following ACC treatment	Shared between IAA and ACC
Reversed Phase Negative	883	260	36	39	6
Reversed Phase Positive	438	154	19	24	7
HILIC Negative	1612	76	11	10	4
HILIC Positive	580	97	13	8	2

Features identified as being different following either treatment are those exhibiting at least a two-fold difference relative to untreated controls in all three biological replicates based on paired univariate analysis.

### Overview of hormone effects on the root metabolome

**Figure 1:**
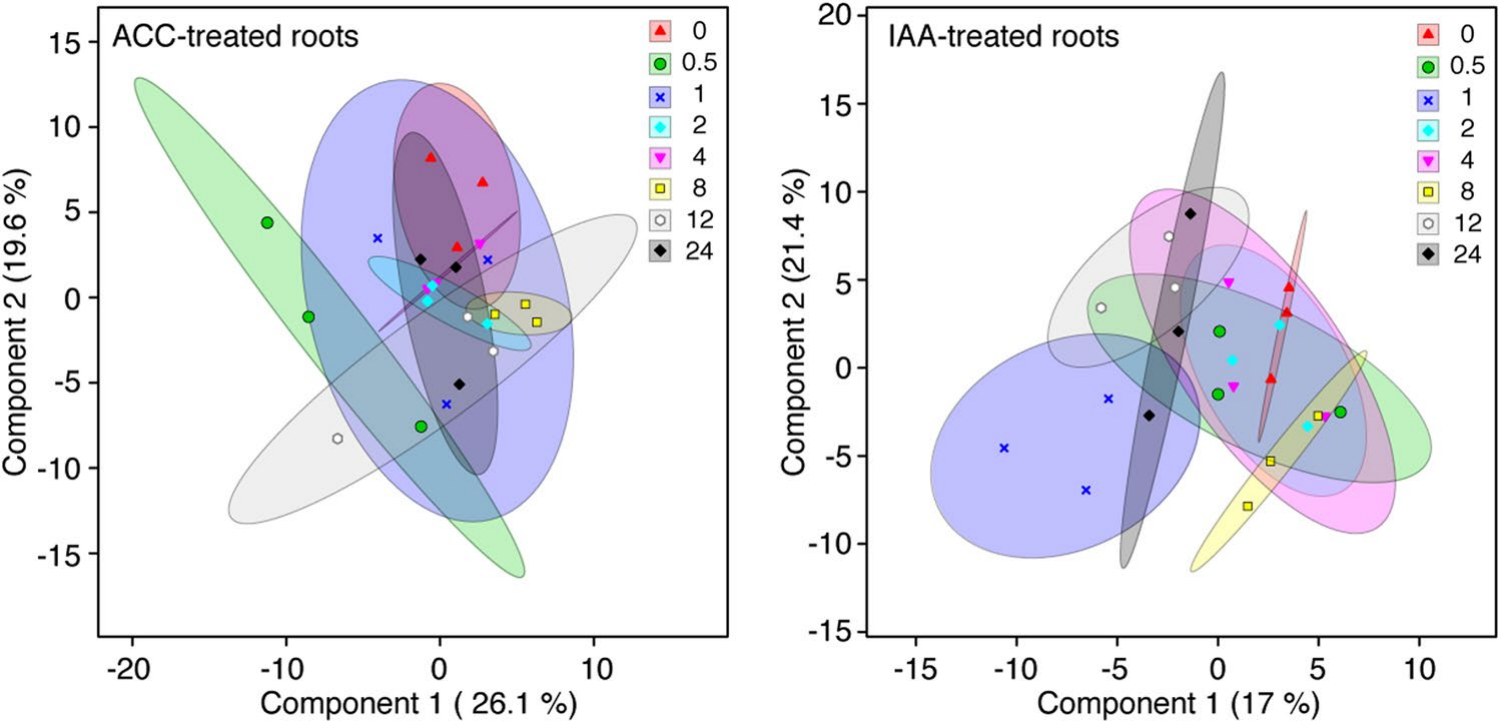
Scores plots derived from sPLS-DA of the reversed phase negative mode metabolomic profiles of ACC- and IAA-treated roots over 24 h. Symbols and colors for each of the eight time points are identified at upper right.

Statistical analysis was used to generate an overview of the metabolome over the time course of exposure to IAA or ACC. Figure 1 shows the scores plots derived from sparse partial least squares-discriminant analysis (sPLS-DA) of the reversed phase negative mode dataset (Dataset S1), which exhibited the largest number of changes for either hormone treatment. This analysis enables the selection of the most discriminative features in the data to help distinguish between individual samples^23^, with the resulting plots providing a powerful visualization, in this case of changes in the root metabolome over time. Panel A provides an overview of the response of the root metabolome after IAA treatment across the 24 h time course utilizing 17.0% and 21.4% of the data in components 1 and 2, respectively. The sPLS-DA shows substantial overlap of the IAA elicited root profiles at the early time points, from 0 to 2 h, but displays a distinct separation of the profiles at 4 h and later. Panel B provides an overview of the metabolome after ethylene elicitation displaying 26.1% and 19.6% of the data in components 1 and 2, respectively. The scores plot shows a rapid and distinct separation at the first sampling point after elicitation, at 30 min, which is transient. The 4 h and later time points separate from the origin but do not display clear distinction among each other. Similar results were observed for the sPLS-DA analysis of datasets generated using the other three analytical methods (Datasets S2–S4).

### Time series analysis reveals specific metabolites that change in abundance following hormone exposure

**Figure 2:**
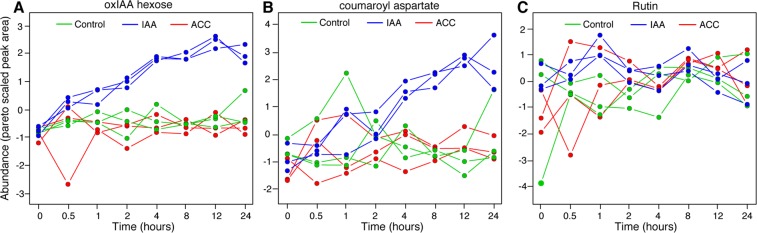
Identification of major patterns associated with time, hormone treatment, and their interactions. The patterns of abundance over time are shown for the two features identified by ASCA, oxIAA hexose (**A**) and coumaroyl aspartate (**B**), and one uncovered by targeted analysis, the flavonoid glycoside rutin (**C**). Note that the x axis is not drawn on a linear scale; this is to allow for better visualization of the data points for early sampling times.

Multivariate statistical methods were used to uncover features displaying distinctive temporal patterns that were driving the profile differences observed in the sPLS-DA score plots. ANOVA Simultaneous Component Analysis (ASCA) uncovered two features (273_352.1028 and 271_278.0659) that exhibited a strong response to IAA treatment over the 24 h time course (Fig. 2). These were putatively identified by fragmentation analysis as 2-oxindole-3-acetic acid-hexose (oxIAA hexose) and coumaroyl aspartate (Table S1). ASCA did not identify any features whose abundance exhibited a distinct trend across the time course in response to exposure to ACC.

The separation of time points in the sPLS-DA indicated that the metabolome was changing over time, even though ASCA only correlated two features with a response to hormone treatment. Because ASCA relies on the presence of sustained trends over time, this suggested that additional features could have exhibited changes that were transient or occurred at nonconsecutive time points. To examine this possibility, the reversed phase negative mode dataset was surveyed for a feature associated with the abundant root flavonoid, quercetin hexose deoxyhexose (rutin). Quercetin has previously been shown to exhibit rapid changes in response to IAA and ACC treatments of roots^14^. The time course profile for rutin did indeed reveal a previously-undetected difference, which was an increase at 1 h in response to IAA relative to the untreated control, present in all three biological replicates (Fig. 2C; blue versus green data points).

A paired univariate approach was therefore applied to all four datasets to identify features that exhibited two-fold differences within each biological replicate at one or more time points, which uncovered approximately 80 additional features of interest for each hormone treatment (Table 1). Analysis of fragmentation patterns and assignment of duplicate features from the same molecular species resulted in 26 putative identifications for compounds in the IAA datasets (Tables 2 and S1) and 20 for ACC (Tables 3 and S1). These metabolites belong to a few distinct classes of metabolites, primarily phenylpropanoids, glucosinolates, and several derivatives of indole. Many of the changes within these classes were transient, with elevated levels detected at a single early time point that then rapidly returned to the levels of untreated time-matched controls, often by the next sampling event. The responses to treatment with IAA or with ACC were also quite distinct, both in terms of the timing and the types of metabolites that exhibited altered levels, with only five metabolites in common between the two datasets. Thus, despite substantial crosstalk between auxin and ethylene at the transcriptional and developmental levels, the short-term response to exogenous exposure results in substantially different responses with regard to the metabolic profiles of root cells.

**Table 2:** Putatively-identified root metabolites exhibiting robust changes in response to auxin.

Metabolite	EMRT	Method	Average log two-fold change after transfer to hormone-containing medium						
			0.5 h	1 h	2 h	4 h	8 h	12 h	24 h
**Indole-containing metabolites including glucosinolates**									
IAA hexose*	306.41_336.1079	RP-Neg	−**5.2**	1.9	−**13.9**	−1.7	1.1	2.1	−1.8
oxIAA hexose	273.02_352.1028	RP-Neg	1.7	1.9	**3.4**	**5.1**	**6.0**	**8.3**	**5.2**
6-hydroxyindole-3-carboxylate dihexose	172.03_500.1401	RP-Neg	−**2.9**	2.6	−2.9	−1.8	1.6	2.0	−1.2
6-hydroxyindole-3-carboxylate hexose	190.42_338.0873	RP-Neg	−**3.1**	1.4	−3.2	−1.7	1.2	1.9	−1.6
4-methoxy-3-indolylmethyl glucosinolate	296.17_477.0638	RP-Neg	−1.7	1.1	−**4.2**	−1.8	1.7	4.1	−1.2
**Phenylpropanoids**									
quercetin hexose hexose*	258.76_625.1406	RP-Neg	1.4	**6.1**	**2.2**	1.3	2.0	−1.1	−1.1
quercetin hexose hexose deoxyhexose	292.71_771.1982	RP-Neg	1.5	**3.7**	1.7	1.7	1.6	−1.1	1.2
quercetin hexose deoxyhexose deoxyhexose	306.01_755.2032	RP-Neg	1.6	**7.6**	2.2	1.1	2.1	1.2	1.2
quercetin hexose deoxyhexose (rutin)	307.99_609.1459	RP-Neg	1.6	**3.5**	1.8	1.3	1.5	−1.2	1.1
quercetin deoxyhexose pentose	314.09_579.135_	RP-Neg	1.6	**4.0**	**2.1**	1.5	1.8	1.2	1.2
quercetin hexose	352.44_463.0873	RP-Neg	1.2	**8.1**	**4.4**	1.9	2.3	−1.5	1.3
isorhamnetin hexose deoxyhexose	335.2_623.1614	RP-Neg	1.4	**3.5**	2.0	1.6	1.7	−1.1	1.3
isorhamnetin hexose	386.17_477.1031	RP-Neg	1.0	**10.6**	**4.2**	1.7	3.6	−1.3	1.2
kaempferol hexose deoxyhexose	329.4_593.151	RP-Neg	1.3	**2.7**	2.0	1.4	1.4	−1.0	1.3
kaempferol hexose deoxyhexose deoxyhexose*	327.01_739.2083	RP-Neg	1.0	**3.8**	**3.2**	1.1	2.0	1.1	1.7
kaempferol deoxyhexose deoxyhexose*	354.4_577.1557	RP-Neg	1.3	2.3	**2.4**	1.2	3.0	−1.1	1.6
kaempferol hexose	379.71_447.0924	RP-Neg	1.3	4.6	**5.7**	2.1	3.6	−1.0	1.4
coumaric acid hexose (isomer a)	265.14_325.092	RP-Neg	−1.0	**5.1**	2.9	−1.1	3.9	−1.4	1.3
coumaric acid hexose (isomer b)	278.71_325.092	RP-Neg	−1.2	**4.0**	1.4	−1.2	4.4	−1.2	−1.4
caffeic acid hexose	232.68_341.0869	RP-Neg	1.0	**6.5**	2.2	−1.4	4.1	−1.4	1.1
coumaroyl aspartate	271.05_278.0659	RP-Neg	1.1	2.4	1.5	**6.2**	**11.6**	**28.9**	**8.9**
**Fatty acids**									
phosphatidylcholine 34:2	170.7_796.523	HILIC-Pos	3.4	**3.6**	−1.1	1.3	−1.7	−1.8	−**2.6**
phosphatidylcholine 34:3	171.23_756.554	HILIC-Pos	4.3	**3.6**	−1.1	1.3	−1.8	−2.2	−**2.6**
phosphatidylcholine 36:5	171.16_780.554	HILIC-Pos	4.8	3.3	−1.1	1.5	−1.7	−1.8	−**2.4**
phosphatidylcholine 36:4	170.85_782.568	HILIC-Pos	4.5	3.9	−1.2	1.4	−1.8	−1.8	−**2.9**
trihydroxyhexadecanoic acid hexose*	481.27_465.2694	RP-Neg	1.9	**8.1**	−1.4	1.4	−1.4	1.7	1.2

Included are all metabolites with features that differed ≥ 2 fold relative to the time-matched control in all three biological replicates at one or more sampling times; values that meet these criteria are shown in bold and are underlined. Values are the average fold change for the three replicates, with positive numbers indicating elevated levels and negative numbers indicating reduced levels relative to untreated controls. Putative identifications are based on fragmentation patterns obtained by MS/MS and MSE, with further details available in Table S1. Asterisks identify the five metabolites common to the auxin and ethylene datasets. RP = reversed phase; HILIC = hydrophilic interaction chromatography.

**Table 3:** Putatively-identified root metabolites exhibiting robust changes in response to ethylene.

Metabolite	EMRT	Method	Average log two-fold change after transfer to hormone-containing medium						
			0.5 h	1 h	2 h	4 h	8 h	12 h	24 h
**Glucosinolates**									
4-methylsulfinylbutyl glucosinolate	73.32_436.0403	RP-Neg	−**2.7**	−1.6	−1.2	−2.1	1.5	4.7	−3.8
7-methylsulfinylheptyl glucosinolate	374.59_478.0873/238.06_478.0875	RP-Neg/HILIC-Neg	−**2.8**	−1.5	−1.1	−1.3	2.2	2.2	**−4.8**
8-methylsulfinyloctyl glucosinolate	432.65_492.103/230.65_492.1032	RP-Neg/HILIC-Neg	−**3.0**	−1.7	−1.2	−1.4	2.6	3.2	**−6.0**
4-methylthiobutyl glucosinolate	233.56_420.0453	RP-Neg	−2.3	−2.6	−1.5	−2.3	1.5	4.0	**−6.8**
5-methylthiopentyl glucosinolate and hydroxy-8-methylsulfinyl octyl glucosinolate (isomer a)	284.19_434.061	RP-Neg	−1.9	−2.4	−1.3	−1.6	1.2	2.1	**−4.2**
hydroxy-8-methylsulfinyl-octyl glucosinolate (isomer b)	244.53_508.098	RP-Neg	−3.6	−1.5	−1.2	−1.2	2.6	2.0	**−3.5**
7-methylthioheptyl glucosinolate	399.01_462.0926	RP-Neg	−1.6	−1.7	−1.0	−1.5	1.3	2.4	**−4.7**
8-methylthiooctyl glucosinolate	454.6_476.1082	RP-Neg	−1.6	−1.6	−1.1	−1.4	1.0	1.9	**−4.5**
4-benzoyloxy *N*-butyl glucosinolate	379.81_494.0789	RP-Neg	−1.4	−2.8	−1.3	−1.7	1.5	3.1	**−5.9**
*N*-methoxyglucobrassicin	340.76_477.0637	RP-Neg	−2.2	−2.7	−1.4	−1.1	1.5	2.8	**−5.4**
**Phenylpropanoids**									
*N*,*N*-9-di(sinapoyl)-spermidine	165.11_558.281	HILIC-Pos	**2.5**	1.5	−1.4	1.3	1.4	−1.2	−1.1
quercetin hexose hexose*	258.76_625.1406	RP-Neg	1.7	1.7	**2.2**	1.2	2.1	1.8	−1.0
quercetin-containing unknown	432.71_835.2083	RP-Neg	1.9	2.6	**2.7**	1.3	2.9	2.3	−1.1
kaempferol deoxyhexose deoxyhexose*	354.4_577.1557	RP-Neg	2.0	1.4	1.3	−1.1	**3.0**	1.4	1.4
kaempferol hexose deoxyhexose deoxyhexose*	327.01_739.2083	RP-Neg	1.4	1.2	2.4	1.1	2.8	**2.6**	1.6
G(8-O4)S(8-5)FA hexose	474.1_759.25	RP-Neg	1.2	1.4	−1.2	1.7	2.0	**2.5**	−1.4
hexose G(8-O-4)FA malate + 238	495.4_789.2605	RP-Neg	1.1	1.2	−1.1	1.1	2.2	**2.5**	−1.3
hexose G(8-O-4) FA malate + 238	470.52_789.2605	RP-Neg	1.4	1.4	−1.1	1.3	2.3	**2.6**	−1.4
**Indole-containing metabolites**									
IAA hexose*	306.41_336.1079	RP-Neg	3.2	−1.3	−1.6	2.4	−1.3	−1.3	−**3.8**
**Fatty acids**									
trihydroxyhexadecanoic acid hexose*	481.27_465.2694	RP-Neg	2.9	2.4	3.6	**3.4**	−**1.0**	−**1.2**	−**1.2**

Included are all metabolites with features that differed ≥ 2 fold relative to the time-matched control in all three biological replicates at one or more sampling times; values that meet these criteria are shown in bold and are underlined. Values are the average fold change for the three replicates, with positive numbers indicating elevated levels and negative numbers indicating reduced levels relative to the untreated controls. Putative identifications are based on fragmentation patterns obtained by MS/MS and MSE, with further details available in Table S1. Asterisks identify the five metabolites common to the auxin and ethylene datasets. RP = reversed phase; HILIC = hydrophilic interaction chromatography. Standard abbreviations are used for oligolignols, with G, S, and FA = guaiacyl units, syringyl units, and ferulic acid, respectively.

### The response of the root metabolome to exogenous IAA has the hallmarks of an effort to maintain auxin homeostasis

#### Immediate response to exogenous auxin

A response to auxin exposure was already detectable in the root metabolome at the first time point, 0.5 h after the start of treatment. Particularly striking at this stage was a substantial (5.18 fold) decline in the levels of **IAA-hexose** relative to the untreated controls (Table 2), followed by an even larger (13.8-fold) reduction at 2 h, one of the largest differences observed for any metabolite in this experiment. This is consistent with very rapid activation of a mechanism for achieving auxin homeostasis through the reversible sequestration and/or inactivation of free IAA (reviewed in^24^ and discussed in^25^), followed by buffering of IAA levels by hydrolysis of hex-IAA as other mechanisms for sequestration, exclusion, or transport come into play. This interpretation is further supported by the further-reduced levels of IAA-hexose at 2 h, which were accompanied by elevated levels of the glycoside of the IAA oxidation pathway product, **oxIAA-hexose**, also discussed further below. An inverse relationship between the levels of these two metabolites has previously been reported^25^. The very early activation of IAA homeostasis mechanisms is also consistent with the finding that free hormone was not observed in either control or IAA-treated roots at any of the sampling times under the conditions used for these experiments.

Transcriptome data indicate that expression of genes associated with the synthesis or hydrolysis of IAA-hexose, specifically two IAA glucosyl transferases (UGT74D1 and UGT84B1^26,27^;) and members of the IRL1 family (ILR1, ILL2, IAR3/ILL4, and ILL5, homologs of which encode glucosyl hydrolase activity in Medicago and rice^28,29^;), is largely unaffected by exposure to auxin under the current conditions (Datafile S5). This suggests that key enzymes remain to be identified or that post-transcriptional processes are responsible for the observed early changes in IAA-glucose levels. On the other hand, the glycosyl hydrolysis reaction is energetically favored and could potentially proceed instantaneously in response elevated levels of auxin, without the need for changes in a specific enzyme activity. Another possibility, which is also consistent with a role for IAA conjugation in maintaining auxin homeostasis, is that the rapid drop in IAA-hexose levels within the first 0.5 h of exposure of roots to auxin reflects activation of transport machinery to rapidly move excess free and/or conjugated hormone out of the root to other sites in the plant or to the rhizosphere. Although transporters able to mobilize IAA-hexose remain to be identified, the transcriptome data show that genes encoding the auxin efflux carrier PIN-FORMED (PIN) proteins, *PIN1*, *PIN3*, and *PIN7*, are rapidly induced (Table 4). The current findings thus suggest the possibility that activation of transport machinery for various forms of auxin may help mediate the early response to abrupt changes in this hormone.

**Table 4:** Transcripts associated with auxin-responsive metabolic processes.

ID	Gene Number	ProbeSet No.	Average SLR change after transfer to hormone-containing medium							
			0 h	0.5 h	1 h	2 h	4 h	8 h	12 h	24 h
**IAA metabolism**, **transport**, **signaling**										
DAO1*	AT1G14130	262653_at	−0.27	0.23	−0.15	0.32	0.03	−**0.72**	−0.47	0.08
DAO2*	AT1G14120	262608_at	0.02	−0.26	−***1.00***	−0.54	−**1.27**	−**1.57**	−**1.28**	−0.55
GH3.1	AT2G14960	266611_at	−0.02	**2.09**	**2.05**	**3.56**	**3.95**	**4.12**	**3.41**	**2.60**
GH3.3*	AT2G23170	245076_at	−0.56	**4.94**	**4.49**	**5.58**	**6.10**	**6.08**	**4.67**	**3.94**
GH3.5	AT4G27260	253908_at	−0.10	**2.97**	**3.13**	**3.89**	**4.00**	**4.49**	**4.12**	**4.01**
GH3.6*	AT5G54510	248163_at	0.00	**2.00**	**1.98**	**2.51**	**2.41**	**2.84**	**2.52**	**2.01**
CYP79B2†	AT4G39950	252827_at	−0.23	0.24	−0.56	−**1.61**	−0.54	−0.15	−0.15	0.04
CYP79B3†	AT2G22330	264052_at	−0.08	−0.17	−0.25	−1.30	−**1.09**	−0.49	−0.24	−0.37
NIT1, NIT2	AT3G44310 AT3G44300	252678_s_at	−0.05	−0.24	−0.22	0.29	−0.16	−**0.73**	−**1.08**	−0.53
UGT74E2*	AT1G05680	263231_at	0.06	−0.20	−**1.22**	−0.57	−**1.16**	−**2.34**	−**2.54**	−1.68
PIN1	AT1G73590	259845_at	−0.02	**1.04**	**1.32**	**1.53**	**1.17**	0.48	0.17	0.34
PIN3	AT1G70940	262263_at	−0.14	**1.33**	**1.20**	**1.27**	**1.53**	**1.31**	**1.17**	**0.57**
PIN7	AT1G23080	264900_at	0.00	**0.89**	**1.40**	**1.76**	**1.67**	**1.29**	**1.34**	**0.83**
ASP4	AT1G62800	262646_at	−0.17	−0.10	−0.06	−0.56	−**0.82**	−0.57	−0.31	−0.02
**Ethylene metabolism**										
ACO1*	AT2G19590	265948_at	0.03	−0.20	0.33	**1.00**	**1.14**	**0.93**	**0.84**	0.68
ACO3*	AT1G12010	264346_at	−0.02	−0.08	0.04	0.47	**0.74**	**1.30**	**0.94**	**0.90**
ACO4	AT1G05010	265194_at	−0.08	0.14	0.00	−0.23	−**0.78**	−0.52	−0.38	0.47
ACS5	AT5G65800	247159_at	0.09	−0.22	−0.20	−0.58	−**0.53**	−0.59	−**0.62**	−0.40
ACS6	AT4G11280	254926_at	−0.05	**1.36**	0.61	**0.97**	0.38	0.00	−**0.99**	0.45
ACS7	AT4G26200	253999_at	−0.17	0.51	−0.33	−0.41	−0.26	−**0.74**	−**0.88**	−0.34
ACS8	AT4G37770	253066_at	0.07	**0.91**	**0.61**	**1.14**	**1.03**	0.65	0.20	0.27
ACS9	AT3G49700	252279_at	0.12	**1.05**	**1.38**	**2.08**	**2.27**	**2.00**	**2.15**	**1.31**
ACS11*	AT4G08040	255177_at	0.05	**1.83**	**1.33**	**1.52**	**1.77**	**0.96**	**0.07**	0.26
**Glucosinolate metabolism**, **including indole glucosinolate biosynthesis**										
BCAT4*	AT3G19710	257021_at	−0.32	0.09	−0.35	0.34	0.24	0.21	**1.19**	**0.75**
CYP79F1, CYP79F2	AT1G16410 AT1G16400	262717_s_at	−0.20	0.39	−0.12	0.12	0.19	−0.03	**0.62**	0.39
CYP79B2†	AT4G39950	252827_at	−0.23	0.24	−0.56	−**1.61**	−0.54	−0.15	−0.15	0.04
CYP79B3†	AT2G22330	264052_at	−0.08	−0.17	−0.25	−1.30	−**1.09**	−0.49	−0.24	−0.37
CYP81F2*	AT5G57220	247949_at	−0.48	−0.26	−0.64	−**0.81**	0.45	−0.40	−0.66	−**1.88**
CYP83A1*	AT4G13770	254687_at	−0.15	0.01	−0.02	0.31	0.34	0.03	**0.96**	0.54
UGT74B1	AT1G24100	264873_at	−0.27	0.29	−0.36	−0.73	−**0.85**	−0.50	−0.19	0.23
**Phenylpropanoid Metabolism**										
**General**										
PAL1	AT2G37040	263845_at	0.01	−0.19	−0.45	−0.59	−0.63	−**0.70**	−0.57	−0.21
PAL2	AT3G53260	251984_at	0.01	−0.12	−0.36	−0.24	−0.33	−**0.80**	−0.63	−0.47
PAL4*	AT3G10340	259149_at	0.06	−0.17	−0.39	−0.84	−**1.05**	0.30	0.02	−0.14
4CL1	AT1G51680	256186_at	0.13	−0.05	−0.43	−0.55	−**0.73**	−0.35	−0.40	−0.04
4CL3*	AT1G65060	261907_at	0.18	−0.34	−0.27	−0.46	−0.61	−**0.79**	−0.30	−0.58
**Hydroxycinnamate/lignin metabolism**										
CCR2	AT1G80820	261899_at	0.01	−0.22	−**0.95**	−0.38	−0.17	−0.33	0.09	−0.21
F5H/ CYP84A1*	AT4G36220	261899_at	−0.17	0.23	**0.66**	−0.23	−0.39	−0.10	0.45	**0.76**
CYP84A4 (F5H activity)	AT5G04330	245710_at	−0.07	−0.25	−0.15	−**0.64**	−**0.70**	−**0.62**	−**0.59**	−0.35
CCOAMT*	AT1G67980	260015_at	−0.50	0.17	−**0.98**	−0.10	0.65	0.54	0.54	−**0.97**
CCOAOMT7*	AT4G26220	253985_at	0.27	−0.29	−0.07	0.13	−0.17	−**1.11**	−**0.97**	−**1.07**
ALDH2C4*	AT3G24503	258140_at	−0.15	0.07	0.12	0.72	**0.88**	0.33	0.30	−0.29
CAD-like	AT1G30760	264527_at	0.00	0.00	0.31	**1.58**	**2.52**	**4.32**	**4.52**	**3.50**
CAD6	AT4G37970	253017_at	0.19	−0.46	−0.89	−0.18	0.77	0.84	**0.79**	**0.88**
CAD8	AT4G39330	252943_at	0.03	0.12	−0.31	0.20	0.15	0.33	**0.66**	**1.50**
**Flavonoid**										
CHS	AT5G13930	250207_at	0.34	−0.53	−0.36	−0.33	−0.45	−**0.72**	0.51	−0.76
CHI*	AT2G43570	260568_at	−0.32	0.26	−**0.94**	0.38	0.53	−0.05	0.15	−0.03
F3H*	AT3G51240	252123_at	0.31	−0.53	−0.55	−**0.82**	−**1.04**	−**1.03**	−0.02	−0.60
F3’H*	AT5G07990	250558_at	0.31	−0.54	−0.50	−0.58	−0.34	−**0.55**	−0.07	−0.54
UGT71C3*	AT1G07260	256053_at	−0.01	−0.36	−0.43	−0.30	0.11	0.43	**0.64**	0.65
**Lipid Metabolism**										
**Fatty acid metabolism**										
FAH1	AT2G34770	267318_at	0.23	−0.16	−0.55	−**0.92**	−**0.88**	−**0.56**	−0.37	−0.48
ACC2*	AT1G36180	256459_at	0.13	−0.29	−0.24	0.04	−0.29	−0.61	−**0.72**	−0.17
BCCP2	AT5G15530	246565_at	0.15	−0.17	0.08	−0.28	−0.45	−0.42	−**0.57**	−0.61
NBRFSP	AT4G13180	254759_at	−0.07	**0.83**	0.53	**1.11**	0.63	−0.26	−0.56	−0.05
**Phosphatidylcholine metabolism**										
PLDALPHA1	AT3G15730	258226_at	0.03	−0.01	0.24	0.13	0.26	0.27	**0.59**	0.32
PLDGAMMA1*	AT4G11850	254847_at	−0.12	0.09	0.10	0.18	**0.70**	0.39	0.33	0.35
PLDDELTA	AT4G35790	253120_at	0.06	−0.15	−0.16	−0.60	−**1.06**	−0.59	−0.44	−0.06
NPC3	AT3G03520	259169_at	−0.11	0.04	−0.31	−**1.20**	−**0.88**	−0.08	−0.03	0.15
NPC4, NPC5*	AT3G03540 AT3G03530	259221_s_at	0.27	−0.13	−0.28	−0.16	0.07	0.25	**0.83**	−0.10
LCAT4	AT4G19860	254547_at	0.04	−0.30	−0.11	−0.28	−**0.69**	−0.17	−0.12	0.10
PLA2-ALPHA	AT2G06925	266500_at	0.12	0.16	0.00	−0.15	0.15	**0.63**	**1.02**	0.66
PLA-IBETA2	AT4G16820	245447_at	−0.03	0.64	0.06	0.43	**1.01**	**0.88**	0.59	0.34
PLP1, PLAIVA*	AT4G37070	246252_s_at	−0.04	−0.37	−0.36	−**1.28**	−**1.20**	−0.69	−0.71	−0.68

Shown are genes having average log two-fold values relative to time-matched controls of ≥ 0.5 or ≤ −0.5 (~1.4 or −1.4-fold differences) in all three biological replicates at one or more sampling times (data from 11,12), with values meeting these criteria shown in bold and are underlined. Positive numbers indicate elevated levels and negative numbers indicate reduced levels relative to the untreated controls. The full list of genes examined for each class with values for each biological replicate are provided in Datafile S5. † denotes genes that participate in indole glucosinolate biosynthesis and thus are listed under two categories. Asterisks identify genes that meet the criteria for change in response to both auxin and ethylene. Enzyme abbreviations are as follows: 4CL (4-coumaroyl coenzyme A ligase), ACC (acetyl-CoA carboxylase), ACO (ACC oxidase), ACS (ACC synthase), ALDH (aldehyde dehydrogenase), ASP (aspartate aminotransferase), BCAT (branched-chain-amino-acid aminotransferase), CAD (cinnamyl-alcohol dehydrogenase), CAT (lecithin-cholesterol acyltransferase-like), CCOAMT (caffeoyl-CoA O-methyltransferase), CCoAOMT7 (caffeoyl-coenzyme A O-methyltransferase), CHI (chalcone isomerase), CHS (chalcone synthase), DAO (DIOXYGENASE FOR AUXIN OXIDATION), F3H (flavanone 3-hydroxylase), F3’H (flavonoid 3’-hydroxylase), FLS (flavonol synthase), GH (GRETCHEN HAGEN), NIT (nitrilase), NPC (non-specific phospholipase C), PAL (phenylalanine ammonia-lyase), PLA (phospholipase A), PLD (phospholipase D), PLP (patatin-like protein), UGT (uridine diphosphate-glycosyltransferase).

A similar early and transient decrease in abundance was observed at 0.5 h for two glycosides of **6-hydroxyindole-3-carboxylate**, approximately 3-fold in each case (Table 2). These compounds are downstream components of the indole glucosinolate pathway, the intermediates of which can be used for IAA synthesis in Brassicaceae^30,31^. The reduced levels may thus reflect a rapid downregulation of indole glucosinolate metabolism to mitigate the *de novo* production of additional IAA. Indeed, a four-fold reduction was observed slightly later, at 2 h, one of two terminal products of this pathway, **4-methoxy-3-indolylmethyl glucosinolate** (Table 2). However, these changes were again not well correlated with changes in the transcriptome. Although transcripts encoding several enzymes of indole glucosinolate metabolism (CYP79B2/3, CYP81F2 and UGT74B1) exhibit decreases, these are first observed at 2–4 h, well after the reduction in the two downstream carboxylates relative to untreated controls. Moreover, no changes were observed for transcripts encoding several other enzymes of aromatic and indole glucosinolate biosynthesis (CYP79A2, CYP83B1, SUR1, UGT74B1, and SOT16) or those required for synthesis of the carboxylate derivatives (CYP71B6 and AAO1) (Datafile S5). Conversely, there were no substantial changes in the levels of aliphatic glucosinolates, despite changes in the transcripts for a number of the corresponding enzymes (BCAT4, CYP79F1/2, CYP83A1). Thus, here too, it appears that post-translational mechanisms may be key to the early response at the level of the metabolome, including transport of glucosinolates to other parts of the seedling^32^ or secretion into the rhizosphere.

Implementation of a distinct response was apparent at the 1 h and 2 h time points, with a sudden, short-lived increase in the levels of a number of different **flavonol glycosides** relative to time-matched controls (Table 2). These included six quercetin glycosides, two glycosides of isorhamnetin (a 3’-methoxylated derivative of quercetin), and four kaempferol glycosides, all of which exhibited a substantial (2–11 fold) and transient (1–2 h) increase. This finding is consistent with previous studies that reported an increase in flavonols and in the quercetin:kaempferol ratio under similar experimental conditions, although it occurred somewhat later in the time course^14^. A glycoside of **coumarate**, a precursor for the synthesis of flavonoids and monolignols, exhibited the same short-lived increase, as did a glycoside of the monolignol intermediate, **caffeic acid**, suggestive of altered flux across the phenylpropanoid pathway, the branches of which are highly interconnected (e.g.^33,34^). Flavonols, and quercetin in particular, are well-established inhibitors of auxin transport^14^. Thus an early strategy for regaining auxin homeostasis could include shutting down the import of exogenous auxin, both from the medium and from the primary site of synthesis in shoots, by raising flavonol levels in the root.

As with the other early responses of the root metabolome to auxin, changes in the transcriptome could not explain the observed accumulation of phenylpropanoids at the 1 and 2 h sampling times. The only phenylpropanoid enzyme for which transcript levels increased during this time frame was the lignin pathway enzyme, ferulate 5-hydroxylase (F5H), which exhibited slightly-elevated levels at 1 h (Table 4; Datafile S5). Previous qRT-PCR analyses of Arabidopsis roots exposed to 1 µM IAA or ACC detected 3- to 4-fold increases in transcripts for several flavonoid genes^14^, but in this prior study roots were grown on higher levels of sucrose, which has been shown to increase expression of pathway enzymes^35^, and to result in more profound root developmental phenotypes in mutants with defects in flavonol synthesis^36^. However, even in this case, expression peaked at the 2 h time point, lagging behind the changes in the abundance of flavonols observed here, similar to what has also been reported for the light response of flavonoids and associated genes in roots^37^. Once again, it appears that immediate/early mechanisms for re-establishing auxin homeostasis largely involve post-transcriptional mechanisms for coordination and reconfiguration of existing metabolic networks. These may also involve long-distance translocation or secretion processes, both well documented in the case of flavonoids^38^.

On the other hand, it appears that changes in gene expression occur at later time points that could contribute to the observed return of the quercetin, kaempferol, coumarate, and caffeic acid conjugates to pre-exposure levels, with transcript levels for a number of enzymes of general phenylpropanoid and flavonoid metabolism decreasing relative to untreated controls starting at 1–2 h (Table 4). Interestingly, Kelch domain-containing F-box proteins have recently been implicated in the ubiquitin-mediated degradation of PAL^39^ and CHS^40^ and transcripts encoding two of the PAL-specific KFBs do exhibit elevated levels in response to auxin (at 1, 4, 8, and 12 h for KFB1 and 1 h for KFB20; Datafile S5). This provides evidence from the transcriptome that post-translational modifications may also help mediate the reduction of flux through the phenylpropanoid pathway to return flavonoid metabolites to pre-exposure levels.

#### Second stage response

The immediate responses of the metabolome appeared to be followed by implementation of two well-established auxin inactivation mechanisms at 2–4 h, both of which persisted through the rest of the sampling times, with peak activity apparent at 8–12 h. This was a unique pattern among the features/metabolites identified in this study, making these the only two uncovered by ASCA, as described above (Fig. 2). The first of the mechanisms was evidenced by the appearance of strongly elevated (3- to 8.5-fold) levels of **oxIAA hexose** (Table 2), the major primary IAA catabolite in Arabidopsis roots^41,42^. This oxidized, inactive form of IAA is generated through the action of two DIOXYGENASE FOR AUXIN OXIDATION (DAO) enzymes (reviewed in^43^). However, contrary to expectations, over the same time period transcripts encoding DAO1 and DAO2 are reduced 2- to 3-fold [signal log ratio (SLR) of −1.00 to −1.57; Table 4]. This suggests that posttranscriptional processes such as changes in activity of this enzymatic machinery may drive auxin inactivation mechanisms, even after 8 or more hours, while changes in gene expression may contribute to the return to pre-exposure conditions.

A second metabolite, **coumaroyl aspartate** (hydroxycinnamoyl aspartic acid), exhibited a very similar pattern of induction, with levels that were substantially higher than in untreated controls at 12 h, among largest changes observed for any metabolite (Table 2). It was also the only phenylpropanoid pathway-derived product that was elevated at 4 h and thereafter, and thus also the only one correlated with elevated transcripts for a cinnamyl-alcohol dehydrogenase (CAD)-like enzyme (at 2–24 h) and two CAD enzymes (at 12–24 h) (Table 4). This compound has been reported in only a limited number of prior studies, notably in roasted coffee and cocoa beans (e.g.^44,45^), as the major metabolite after elicitor treatment of cell suspension cultures of European beech^46^, and in cell suspension cultures derived from Arabidopsis cotyledons^47^. It is also one of many compounds proposed to accumulate upon perturbation of the lignin pathway, which derives from *p-*coumarate in phenylpropanoid metabolism^48^. Thus it is possible that accumulation of coumaroyl aspartate is related to the earlier shift of flux into flavonoids and monolignols. Another possibility is that the higher levels of this compound are indicative of mobilization of another primary auxin inactivation mechanism, conjugation to aspartate. Indeed, the associated transcriptome date show a rapid and strong induction of transcript levels for several *GRETCHEN HAGEN 3 (GH3)* genes (*GH3*.*1*, *3*.*3*, *3*.*5*, and *3*.*6;* Table 4), which encode key enzymes for conjugation of aspartate, as well as other amino acids, to IAA^49^. Defining the position of coumaroyl aspartate in the metabolic network should provide new insights into the role of this compound in the response to auxin and other perturbations.

#### Transition to a new state of homeostasis

Changes in one additional class of metabolites characterized the response to auxin, including at the final, 24 h, time point, a reduction in several forms of **phosphatidylcholine** (PC). These metabolites represent the major class of phospholipids in eukaryote membranes, serving key roles as both structural and signaling molecules. Two forms, 34:2 and 34:3, exhibited a robust elevation early in the time course, at 2 h, together with an 8-fold increase in the fatty acid derivative, trihydroxyhexadecanoic acid. The PCs were then detected at reduced levels at 24 h, together with two 36-carbon PCs, 36:4 and 36:5. Changes in membrane lipid composition have been shown to mediate localization and activity of several PIN efflux carriers, and genes involved in this process are emerging from a number of different studies, including in roots^50^. These include the phospholipases, PLP1 and NPC3, that are proposed to have a role in processes linked to auxin signaling, including lateral root development and modification of root architecture in response to phosphate starvation^51,52^. A number of genes with roles in PC metabolism exhibit fluctuations in expression starting with the 2 h time point (Table 4), indicating that transcriptional changes may, at least in part, mediate the observed changes in PC levels. These findings also suggest that modifications in lipid membrane composition may contribute to maintenance of auxin homeostasis in the face of continued elevated levels of the hormone.

### Ethylene induces a distinct response at the level of the metabolome

Changes in the root metabolome in response to ACC and the resulting elevated levels of ethylene were substantially different from those observed with auxin, despite the considerable crosstalk occurring between these hormones (Table 3). Even the few metabolites that were common to both responses exhibited distinct temporal patterns. A unique feature of the ethylene response was a substantial reduction in a large number of **glucosinolates** (Table 3). This was initially apparent already at 0.5 h, with an approximately 3-fold reduction in three aliphatic forms. The two octyl forms were also substantially reduced again at 24 h, together with five other aliphatic forms and two aromatic glucosinolates, all between 3.5 and 7-fold lower than in untreated tissues. A number of these metabolites were previously reported to be abundant in roots of mature plants^53^. The reduction in the levels of these compounds suggests that the response to ACC includes a change in the rate of flux through all three pathways of glucosinolate biosynthesis. Analysis of the corresponding transcriptome data^12^ showed a striking lack of correlated suppression of expression of genes encoding enzymes in these pathways over the 24 h time course, with the only substantial differences with the time-matched controls being increases at various sampling times, rather than the expected immediate decreases, in several enzymes of aliphatic (BCAT4, CYP83A1, SOT18) and aromatic (CYP83B1) metabolism (Table 5; Datafile S5). There was also an increase in transcripts for an enzyme of indole glucosinolate biosynthesis (CYP81F2) at 12 and 24 h, with no corresponding change in the corresponding metabolites. Once again, it appears that posttranscriptional mechanisms control the early response of a major pathway of specialized metabolism to hormone exposure.

**Table 5:** Transcripts associated with ethylene-responsive metabolic processes.

ID	Gene Number	ProbeSet No.	Average SLR change after transfer to hormone-containing medium							
			0 h	0.5 h	1 h	2 h	4 h	8 h	12 h	24 h
**IAA metabolism**, **transport**, **signaling**										
DAO1*	AT1G14130	262653_at	−0.34	0.17	−0.26	−0.03	−0.50	−**0.82**	−**0.59**	−0.17
DAO2*	AT1G14120	262608_at	−0.09	−0.21	−0.27	−0.23	−**1.03**	−**1.48**	−**1.49**	−0.84
GH3.3*	AT2G23170	245076_at	−0.36	0.98	−0.14	−0.17	0.16	0.48	0.30	**0.82**
GH3.6*	AT5G54510	248163_at	0.26	−0.39	−0.50	−0.46	**−1.22**	−0.50	−0.35	−0.42
CYP71A13	AT2G30770	267567_at	0.26	0.50	−**0.96**	−0.42	−0.13	−0.43	−**0.79**	0.17
UGT74E2*	AT1G05680	263231_at	0.16	0.59	−0.98	−**1.15**	−**2.53**	−**2.84**	−**2.57**	−**1.90**
**Ethylene Metabolism**										
ACO1*	AT2G19590	265948_at	0.16	0.38	**1.41**	**1.72**	**2.23**	**1.96**	**1.73**	**1.54**
ACO2	AT1G62380	260637_at	−0.03	0.35	0.41	**0.53**	**1.02**	**1.32**	**1.41**	**1.02**
ACO3*	AT1G12010	264346_at	0.04	0.24	**0.72**	**0.80**	**1.56**	**2.49**	**2.30**	**1.48**
ACO5	AT1G77330	246390_at	−0.13	−0.32	**1.11**	**1.19**	**0.70**	**1.00**	**1.19**	**0.88**
ACS11*	AT4G08040	255177_at	−0.41	**0.80**	−0.60	−0.47	−0.15	0.18	0.18	0.14
**Glucosinolate Metabolism**										
BCAT4*	AT3G19710	257021_at	0.49	−0.19	−0.38	−0.79	−0.03	0.48	**1.59**	0.101
CYP81F2*	AT5G57220	247949_at	0.13	0.97	0.00	−0.67	0.93	1.81	**1.95**	**1.65**
CYP83A1*	AT4G13770	254687_at	**0.71**	−0.32	0.16	−0.50	0.36	0.60	**1.32**	−0.07
CYP83B1	AT4G31500	253534_at	−0.52	0.43	**0.60**	−0.29	0.19	0.20	0.17	0.22
SOT18	AT1G74090	260385_at	0.13	0.04	0.08	−0.43	0.35	0.31	**0.65**	−0.01
**Phenylpropanoid Metabolism**										
**General**										
PAL4*	AT3G10340	259149_at	−0.07	0.28	−0.43	−0.37	−**0.69**	−0.56	−0.78	−0.60
4CL3*	AT1G65060	261907_at	0.44	−0.34	−0.20	−0.09	−0.38	−0.35	−0.27	−**0.58**
**Hydroxycinnamate/lignin metabolism**										
F5H, CYP84A1*	AT4G36220	261899_at	−0.14	−0.50	0.01	−0.39	−0.38	−0.67	−0.35	−**0.85**
CCOAMT*	AT1G67980	260015_at	−0.17	1.40	−0.40	0.48	**1.20**	**2.31**	**2.08**	**2.76**
CCOAOMT7*	AT4G26220	253985_at	0.20	−0.44	0.47	0.63	0.48	**0.82**	**1.00**	0.25
ALDH2C4*	AT3G24503	258140_at	−0.02	0.51	0.38	0.48	**0.80**	0.37	0.52	0.29
OMT1	AT5G54160	248200_at	0.08	0.06	**0.60**	**0.68**	**0.78**	**0.72**	**0.73**	0.27
**Flavonoid**										
CHI*	AT2G43570	260568_at	0.12	0.78	−**0.78**	−0.28	0.37	0.09	0.12	**1.02**
F3H*	AT3G51240	252123_at	0.44	−0.60	−0.34	0.00	−0.48	−0.26	−0.03	−**0.73**
F3’H*	AT5G07990	250558_at	0.64	−0.72	−0.22	−0.26	−0.58	−**0.65**	−0.46	−**1.15**
FLS1	AT5G08640	250533_at	**0.60**	−0.48	−0.23	0.07	−0.08	−0.03	0.18	−0.67
UGT71C3*	AT1G07260	256053_at	0.02	0.16	0.14	0.18	0.38	**0.79**	**1.16**	0.88
**Lipid Metabolism**										
**Fatty acid metabolism**										
ACC2*	AT1G36180	256459_at	0.41	−0.13	−0.20	−0.19	−0.52	−0.57	−**0.75**	−0.62
**Phosphatidylcholine metabolism**										
PLDEPSILON	AT1G55180	259657_at	0.09	0.16	−0.25	0.12	−0.10	0.04	−0.07	−**0.64**
PLDGAMMA1*	AT4G11850	254847_at	0.24	0.87	0.12	0.12	0.64	0.72	**0.84**	0.73
PLDDELTA	AT4G35790	253120_at	0.01	−0.02	−0.07	−0.34	−**0.87**	−0.51	−0.60	−0.45
NPC4, NPC5*	AT3G03540 AT3G03530	259221_s_at	0.31	−0.19	−0.33	−0.39	−0.47	−**0.98**	−**1.17**	−**1.46**
PLP1, AtPLAIVA*	AT4G37070	246252_s_at	0.07	0.45	0.10	−0.64	−**0.92**	−0.43	−0.75	−0.43

Shown are genes having average log two-fold values relative to time-matched controls of ≥ 0.5 or ≤ −0.5 (~1.4 or −1.4-fold differences) in all three biological replicates at one or more sampling times (data from 11,12), with values meeting these criteria shown in bold and are underlined. Positive values indicate elevated levels and negative values indicate reduced levels relative to untreated controls. The full list of genes examined for each class with values for each biological replicate are provided in Datafile S5. Asterisks identify genes that meet the criteria for change in response to both auxin and ethylene. Enzyme abbreviations are as defined for Table 4.

The other major group of compounds characterizing the response to ethylene were products of phenylpropanoid metabolism. These included elevated levels of three **flavonol glycosides**, all of which were also found to change in response to auxin, but in this case with quite different temporal patterns that spanned the 2–12 h time points; an unknown **quercetin-containing metabolite** was also elevated in response to ACC (Table 3). Several genes of central flavonoid metabolism exhibit differential expression at various time points, although not in a coordinated or sustained manner consistent with the observed elevated levels of flavonols observed response to ACC exposure. However, transcripts encoding flavonoid 3’-hydroxylase (F3’H), which controls flux between kaempferol and quercetin, were downregulated at the 8 h time point, consistent with the shift from quercetin to kaempferol-containing metabolites (Table 5). An overall elevation in flavonol levels at 12 h of treatment with ACC, accompanied with an increase in the kaempferol:quercetin ratio, was also previously reported based on staining with DPBA^14^. The only other flavonoid genes with altered expression are a flavonol glycosyltransferase (UGT71C3) and *O*-methyltransferase (OMT1) with elevated levels late in the time course (8–24 h), the former potentially contributing to the enhanced synthesis of the two kaempferol glycosides (Table 3). As mentioned above, although previous studies have detected increases in expression of several flavonoid genes in response to treatment with 1 µM ACC^14^, this is likely attributable to the higher levels of sucrose used in those experiments.

The step-wise changes in glucosinolate and phenylpropanoid products over the 24 h of exposure to ACC points to a gradual shift in flux across the major pathways of specialized metabolism as an initial response to elevated ethylene. The inverse relationship between these two classes of metabolites is consistent with the previous reports of crosstalk between these pathways. However, those cases were the reverse what was observed here, with elevated levels of glucosinolates driving repression of phenylpropanoid metabolism, driven in part by the aldoxime- and MED5-mediated degradation of PAL^54^. Although the changes in the metabolome were again not correlated with altered levels of transcripts encoding pathway enzymes, expression of several PAL KFB’s and the CHS KFB were enhanced, particularly at later time points. Overall, it appears that post-transcriptional processes, including modulation of pathway flux through post-translational control of enzyme levels as well as long-distance transport or secretion of metabolites, may be at play.

Another unique aspect of the ethylene response was an approximately 2.5-fold increase in the levels of three **oligolignols** at 12 h, products of the lignin branch of phenylpropanoid metabolism that are characteristic cell wall components^55^. This latter change is consistent with the alterations in root hair initiation and elongation that occur during the 24 h of ACC exposure, which has also been shown to be accompanied by changes in expression of numerous genes associated with cell wall biogenesis, biosynthesis, and organization^12^. Among these *PAL4*, *4-coumarate:CoA ligase 3* (*4CL3)*, *ferulate 5-hydroxylase (F5H)*, and *CAD8* exhibited reduced levels at various time points, counter to expectations for enhanced oligolignol synthesis (Table 5; Datafile S5) However, several other genes specific to lignin metabolism, *caffeoyl-CoA O-methyltransferase (CCOAMT)*, *caffeoyl-coenzyme A O-methyltransferase (CCoAOMT7)*, *ALDH2C4*, and *OMT1*, were substantially higher, in some cases for sustained periods, indicative of a contribution from transcriptional control mechanisms to changes in oligolignol metabolism observed during the latter part of the time course.

Previous experiments have shown that ethylene positively regulates auxin synthesis^21^. Although free IAA was not detected, **IAA-hexose**, which exhibited reduced levels at 0.5 h of IAA exposure, was also significantly reduced at 24 h in response to ACC. In this case, the difference was preceded by changes in the expression of two *DAO* genes (Table 5), suggesting the possibility that ethylene alters expression of genes contributing to IAA homeostasis via oxidation. However, as with the response to IAA, no substantial changes were observed in transcripts encoding known IAA glycosylase or hydrolase enzymes (Datafile S5). Moreover, changes in the transcripts encoding GH3.3 and 3.6, CYP71A13, and UGT74E2 were not reflected in altered levels of the corresponding IAA metabolites. These observations suggest the possibility of yet-to-be-identified degradation processes or transport/secretion mechanisms specific for IAA conjugates.

## Discussion

The current study represents the first comprehensive analysis of the early response of the root metabolome to auxin and ethylene, phytohormones with well-established synergistic and antagonistic effects on root development. High-resolution data generated using a global untargeted LC-MS approach showed that, in the presence of exogenous IAA and ACC, seedling roots exhibit a series of reproducible and substantial (2-fold or greater) changes to a limited number of classes of metabolites, with the majority of changes being evident within small windows of time. The analysis provided evidence that three distinct strategies are used in response to elevated auxin, one following the other, as plants seek to regain homeostasis in the face of high external levels of the hormone. The compounds exhibiting substantial changes belonged predominantly to three classes: phenylpropanoids, fatty acids, and derivatives of IAA. A distinct response was observed for elevated levels of ethylene that was characterized primarily by changes in glucosinolate and flavonoid levels with minimal overlap with those observed to change in response to IAA. There is evidence in the transcriptome for effects of both hormones on ACC oxidase (ACO) and ACC synthase (ACS) gene expression (Tables 4 and 5) and thus also crosstalk of auxin on ethylene metabolism. However, ACC was not detected in roots under the conditions used in these experiments and these were also not designed to detect volatile compounds such as ethylene. A reduction in IAA-hexose levels at 24 h were the one evidence of crosstalk with IAA signaling pathways.

The finding that altered levels were detected for only a subset of compounds within a particular pathway indicated that certain metabolites are particularly indicative of change in network flux, possibly due to small steady-state pools or short half lives. These compounds may represent additional “sensor” metabolites or biomarkers that can provide insights into the metabolic status of the system as a whole^56^. It is of note that previously-described sensor molecules (e.g., free IAA, L-phenylalanine, naringenin chalcone, eriodictyol, and several glucosinolates including 7-methylthioheptyl and 4-benzyloxybutyl glucosinolates and sinapoyloxy conjugates) did not consistently display differences in the short-term auxin/ethylene response even where there was evidence of altered pathway flux. Thus additional compounds such as those identified in the current study may prove useful, perhaps especially for tracking dynamic responses to biotic or abiotic stress.

Another consistent finding is that changes in the metabolome were not well correlated with previously-published transcriptomics and proteomics datasets for matching tissues. Although the majority of changes at the transcript level were also transient, rapidly returning to the levels present in time-matched controls^11,12^, there was little evidence that these were responsible for the observed changes in the metabolome except possibly in the case of auxin-mediate changes in phosphatidylcholine metabolism and ethylene-induced changes in lignin biosynthesis. For both hormones, the largest differentially-expressed clusters were comprised of known auxin- and ethylene-responsive genes, genes related to RNA and DNA processes, and cell wall biogenesis and organization, among a few others; none were related to specialized metabolism.

This was also true for proteomic profiles generated for the response of roots to auxin at 0.5 and 2h^57^ and at 8, 12, and 24h^58^ under similar or identical conditions, respectively. Although the top auxin-responsive proteins were not related to metabolic processes in either of these studies, GO analysis of the early time points did show evidence of changes in the proteome related to organization of transporters and the cytoskeleton. Profiles of metabolites present in the root exudates of 5–6 week-old hydroponically-grown plants are consistent with secretion as one mechanism for rapidly reducing the levels of specific metabolites, including phenylpropanoids and glucosinolates, as well as other indole-containing compounds^59,60^. A similar outcome has recently been reported in whole seedlings exposed to 1 µM auxin for 3 h, where the majority of differently-expressed proteins were also not correlated with changes in the corresponding transcripts^61^.

This outcome is not particularly surprising in the context of the growing number of studies, including in plants, suggesting that immediate changes in metabolic activity and flux are controlled largely at the post-transcriptional and even post-translational levels (reviewed in^62^). In fact, it has recently been shown that the response to auxin is essentially instantaneous, with elongation of primary roots inhibited within 30 s of exposure to auxin^63^. Even at later time points in the current experiment, it consistently appeared that the response relied on rewiring or mobilizing existing systems through posttranslational mechanisms, an observation for which there is substantial precedent^64^.

## Conclusion

Correlating changes in the metabolic status of plant cells with the underlying mechanistic processes will require extending the elucidation of changes in network flux to the pathways of specialized metabolism, including those that occur immediately following stresses that disrupt equilibrium. It will also require accounting for redistribution, not only of metabolites^32,65,66^, but likely also microRNAs, transcripts, and proteins, within the plant via long-distance transport^67,68^, as well as secretion or volatilization of metabolites away from the plant itself. So, too, must there be information at the level of the individual protein components, including reorganization of intracellular interaction networks and localization. Even the role of post-translational modifications, as in the case of the phenylpropanoid and flavonoid pathways, is just beginning to be uncovered^39,40,54,69^. Technologies to address these questions in a comprehensive manner are increasingly within reach and will help write a new understanding of the processes by which cells execute re-equilibration of their metabolic status, both as part of immediate responses and to achieve new long-term steady states.

## Methods

### Plant growth and hormone treatment

Arabidopsis tissue preparation and hormone treatment were performed as described previously^11,12^. Briefly, Arabidopsis Col-0 seedlings were grown on nylon filters (03-100/32; Sefar Filtration) overlaid on solid MS medium supplemented with 1.0% sucrose. After stratification for 48 h at 4 °C, seeds were germinated under 100 µmol m^−2^ s^−2^ continuous cool white light at 24 °C. On the fifth day after germination, the filters were transferred to MS medium with or without 1 µM IAA (Sigma) or 1 µM ACC (ACROS Organics). Root samples for metabolite analysis were collected by excising the roots just below the root/shoot junction and immediately freezing in liquid nitrogen, followed by storage at −80 °C. Sample collection was performed at 0, 0.5, 1, 2, 4, 8, 12 and 24 h after initial transfer. The time course was performed in triplicate, resulting in three biological replicates for each time point and each hormone/control treatment. The only exception was the 2 h control sample for replicate one, in which the sample mass was insufficient for reliable analysis.

### Metabolite extraction

Root samples were collected in 2 mL polypropylene tubes containing stainless steel beads (2.3 mm diameter, Small Parts, Logansport, IA), immediately flash frozen, and then ground to a fine powder through agitation in a Harbil 5 G paint shaker (Fluid Management Inc., Wheeling, IL) for three cycles of 30 sec each. Samples were submerged in liquid nitrogen after each shaking cycle to ensure the tissue remained frozen during the pulverization process. Metabolite extraction was performed in 99% methanol (Spectrum Chemicals, New Brunswick, NJ) with 1% acetic acid (Sigma, St. Louis, MO) at a ratio of 100 mg tissue/mL acidified methanol. Samples were mixed by vortexing, sonicated for 5 min, and centrifuged at 20,000 × g for 10 min to pellet the insoluble material. The supernatant was removed and a second extraction identical to the first was performed. The extracts were combined and two 250 µL aliquots were taken to dryness in a centrifugal concentrator under vacuum. The dried residue was dissolved in 125 µL of a 9:1 ratio of 0.1% formic acid in water:acetonitrile with 10 ng/µL of acetaminophen (Sigma-Aldrich, St. Louis, MO) for reversed phase analysis or 125 µL of 50:50 ratio of 0.1% formic acid in water:acetonitrile for HILIC analysis. Samples were sonicated for 10 min, centrifuged at 14,000 × g for 10 min to pellet insoluble material, and transferred to a LC-MS vial for analysis.

### UPLC/MS analyses

Sample analysis was performed using a Waters Acquity I-Class UPLC coupled with a Waters Synapt G2-S HDMS quadrupole-time of flight mass spectrometer (Waters Corp., Milford, MA, USA) in centroid mode utilizing randomized technical injections, which were performed in triplicate for each sample. The lock mass reference sprayer infused leucine-enkephalin (1 ng/µL, Waters Corp., Milford, MA) at 5 µL/min with a MS scan frequency of 20 s.

An orthogonal chromatographic approach was used for the untargeted analyses, as described previously^70^. Reversed phase analyses utilized a UPLC BEH C18 column (1.7 µm, 2.1 mm × 50 mm, Waters Corp., Milford, MA) maintained at 35 °C with a flow rate of 200 µL/min and a 15 min gradient from mobile phase A (0.1% aqueous formic acid) to mobile phase B (acetonitrile with 0.1% formic acid). The following gradient conditions were used: isocratic at 1% B (0–1 min), followed by linear gradient to 30% B (1–7 min), to 95% B (7–12 min), isocratic at 95% B (12–12.5 min), followed by hold at initial conditions (13–15 min).

HILIC analysis was carried out on a UPLC BEH Amide Column (1.7 µm, 2.1 mm × 50 mm, Waters Corp., Milford, MA) maintained at 45 °C with flow rate of 400 µL/min and a 10 min gradient from mobile phase B (acetonitrile with 0.1% formic acid) to mobile phase A (0.1% aqueous formic acid). Gradient conditions were isocratic at 99% B (0–0.5 min), a linear gradient to 50% B (0.5–7 min), return to 99% B (7.0–7.1 min), and re-equilibration at the initial condition of 99% B (7.1–10 min).

Column eluents were ionized by electrospray in both positive and negative modes with a cycle time of 0.20 s and a scanned mass range of 50–1800 m/z. For reversed phase separation, the parameters for positive ion mode were source temperature 55 °C, capillary voltage 3.0, cone voltage 40, source offset 30 V, desolvation temperature 350 °C, cone gas 50 L/h, desolvation gas 500 L/h, and nebulizer gas 6.0 bar; parameters were the same for negative ion mode except that source temperature was 125 °C, capillary voltage 2.2, and source offset 80 V. For HILIC, the parameters for positive ion mode were source temperature 120 °C, capillary voltage 3.0, cone voltage 30, source offset 80 V, desolvation temperature 500 °C, cone gas 50 L/h, desolvation gas 600 L/h and nebulizer gas 6.0 bar; parameters for negative ion mode were the same except that capillary voltage was 2.2.

In addition to the analyses described above, a master mix of all samples was analyzed with each chromatography and ionization combination utilizing MS^E^ mode mass spectrometry at different collision energies (10, 20, 30, 40 V). Targeted MS/MS replicating the initial analysis conditions was performed on features found to differ between experimental and control samples to obtain fragmentation patterns for compound identification.

### Peak identification and data processing

Raw data files (*.raw) were converted to the NetCDF format using DataBridge (Waters Corp., Milford, MA). Chromatogram alignment, peak detection, and peak integration were performed in the R statistical programing environment^71^ using the XCMS package^72^ with the following processing parameters: centWave peak detection, 15 ppm mass deviation, peak width (s), 5 min/40 max, signal/noise ratio 2, noise 999, and lockMass adjustment applied (Datasets S1–S4). The XCMS data set was deconvoluted using RamClustR^22^, which identified and merged redundant features resulting from naturally-occurring isotopes, adducts, and in-source fragmentation, before averaging all technical replicates.

sPLS-DA and ASCA were performed on the reduced dataset using the time-series module of the web-based program MetaboAnalyst^73^. A univariate statistical approach was used to discover additional features that exhibited at least a two-fold change in all three biological replicates at one or more time points. Comparison with calculations performed using a 1.5, 2.0 and 2.5-fold cutoff showed that the two-fold criterion was sufficiently high to select against noise, while still uncovering a substantial number of changes. Putative metabolite identities were assigned with the aid of MS/MS and MS^E^ fragmentation patterns, commercial standards, and online databases, including ReSpect^74^, Metlin^75^, MassBank^76^ and KNApSAcK^77^. The details of each metabolite assignment are available in the supplementary materials (Table S1). Data mining for the specific metabolites rutin, IAA and ACC was performed by searching the detected masses of the features in the XCMS positive datasets for masses corresponding to [M + H]^+^ ions and the negative XCMS datasets for masses corresponding to [M − H]^−^ ions. Features were not observed that corresponded to either IAA or ACC, while the details regarding the assignment of rutin are included in Table S1.

### Transcriptome data analysis

Microarray datasets for root samples generated under identical conditions^11,12^ were examined for changes in the expression of genes associated with the synthesis, inactivation, or transport of hormone-responsive metabolites, identified from the KEGG database^78^ or the recent literature (e.g., in the case of members of the GH, DAO, and PIN families). Results for genes exhibiting a SLR of ≥ 0.5 or ≤ −0.5 in all three biological replicates for at least one time point are presented in Tables 4 and 5, with the data for all examined genes provided in Datafile S5.

## Supplementary information

Supporting Information.
